# Environmental suitability for *Lutzomyia longipalpis* in a subtropical city with a recently established visceral leishmaniasis transmission cycle, Argentina

**DOI:** 10.1590/0074-02760170056

**Published:** 2017-10

**Authors:** Pablo Berrozpe, Daniela Lamattina, María Soledad Santini, Analía Vanesa Araujo, María Eugenia Utgés, Oscar Daniel Salomón

**Affiliations:** 1 Ministerio de Salud de la Nación, Instituto Nacional de Medicina Tropical, Puerto Iguazú, Misiones, Argentina; 2 Ministerio de Ciencia, Tecnología e Innovación Productiva, Consejo Nacional de Investigaciones Científicas y Tecnológicas, Buenos Aires, Argentina; 3 Ministerio de Salud de la Nación, Centro Nacional de Diagnóstico e Investigación en Endemo-Epidemias, Administración de Laboratorios e Institutos de Salud, Buenos Aires, Argentina; 4 Universidad Nacional del Nordeste, Facultad de Ciencias Exactas, Naturales y Agrimensura, Corrientes, Argentina; 5 Red de Investigación de la Leishmaniasis en Argentina, Buenos Aires, Argentina

**Keywords:** Lutzomyia longipalpis, leishmaniasis, environmental suitability, Argentina

## Abstract

**BACKGROUND:**

Visceral leishmaniasis (VL) is an endemic disease in northeastern Argentina including the Corrientes province, where the presence of the vector and canine cases of VL were recently confirmed in December 2008.

**OBJECTIVES:**

The objective of this study was to assess the modelling of micro- and macro-habitat variables to evaluate the urban environmental suitability for the spatial distribution of *Lutzomyia longipalpis* presence and abundance in an urban scenario.

**METHODS:**

Sampling of 45 sites distributed throughout Corrientes city (Argentina) was carried out using REDILA-BL minilight traps in December 2013. The sampled specimens were identified according to methods described by Galati (2003). The analysis of variables derived from the processing of satellite images (macro-habitat variables) and from the entomological sampling and surveys (micro-habitat variables) was performed using the statistical software *R.* Three generalised linear models were constructed composed of micro- and macro-habitat variables to explain the spatial distribution of the abundance of *Lu. longipalpis* and one composed of micro-habitat variables to explain the occurrence of the vector.

**FINDINGS:**

A total of 609 phlebotominae belonging to five species were collected, of which 56% were *Lu. longipalpis*. In addition, the presence of *Nyssomyia neivai* and *Migonemya migonei*, which are vectors of tegumentary leishmaniasis, were also documented and represented 34.81% and 6.74% of the collections, respectively. The explanatory variable normalised difference vegetation index (NDVI) described the abundance distribution, whereas the presence of farmyard animals was important for explaining both the abundance and the occurrence of the vector.

**MAIN CONCLUSIONS:**

The results contribute to the identification of variables that can be used to establish priority areas for entomological surveillance and provide an efficient transfer tool for the control and prevention of vector-borne diseases.

Visceral leishmaniasis (VL) is a disease caused by the parasite *Leishmania infantum* (syn. *chagasi*) (Kinetoplastida: Trypanosomatidae). In Argentina, VL is transmitted in urban environments with the domestic dog as the main reservoir species and *Lutzomyia longipalpis* (Diptera: Phlebotominae) as the main vector (Lainson & Rangel 2005).

*Lu. longipalpis* is a species capable of colonising urban environments, with greater activity during the crepuscular hours. It presents a heterogeneous distribution in space and has areas of high abundance and low abundance, as well as areas where the vector is absent (Fernández et al. 2010, Salomón et al. 2015). In Argentina, the first autochthonous human case of VL was detected in the city of Posadas (Misiones province) in 2006, with the presence of *Lu. longipalpis* in the backyard of the site where the index case was detected (Salomón et al. 2008). In December 2008, after the report of canine VL in Corrientes province, which is located south of Misiones, *Lu. longipalpis* dispersion was recorded in the city of Corrientes (Salomón et al. 2009).

The presence and abundance of *Lu. longipalpis* are associated with micro-habitat variables (recorded at the trap site), such as the presence of farmyard animals, dogs, and asphalt, and with macro-habitat variables (recorded at a homogeneous buffer area) calculated from the processing of satellite images (Costa et al. 2005, de Andrade et al. 2014) according to the normalised difference vegetation index (NDVI) (Fernández et al. 2010, Santini et al. 2015).

In recent years, there has been a significant increase in studies that have examined the relationship between health and environmental factors derived from remotely sensed (RS) data. The use of satellite images allows the identification of environmental factors that affect the biology of disease vectors and when supplemented with epidemiological data, can provide information for monitoring and mapping the risk of several diseases (Beck et al. 2000). Entomological risk mapping based on RS information and field-collected data is a useful tool for leishmaniasis monitoring, decision-making and implementation of vector control and prevention strategies (Salomón et al. 2011).

The objective of the present study was to evaluate the micro- and macro-habitat variables that explain the spatial distribution of *Lu. longipalpis* abundance and presence in a recently colonised urban scenario in a subtropical region in order to develop a strategy to prioritise areas for vector surveillance.

## MATERIALS AND METHODS

**Fig. 1:**
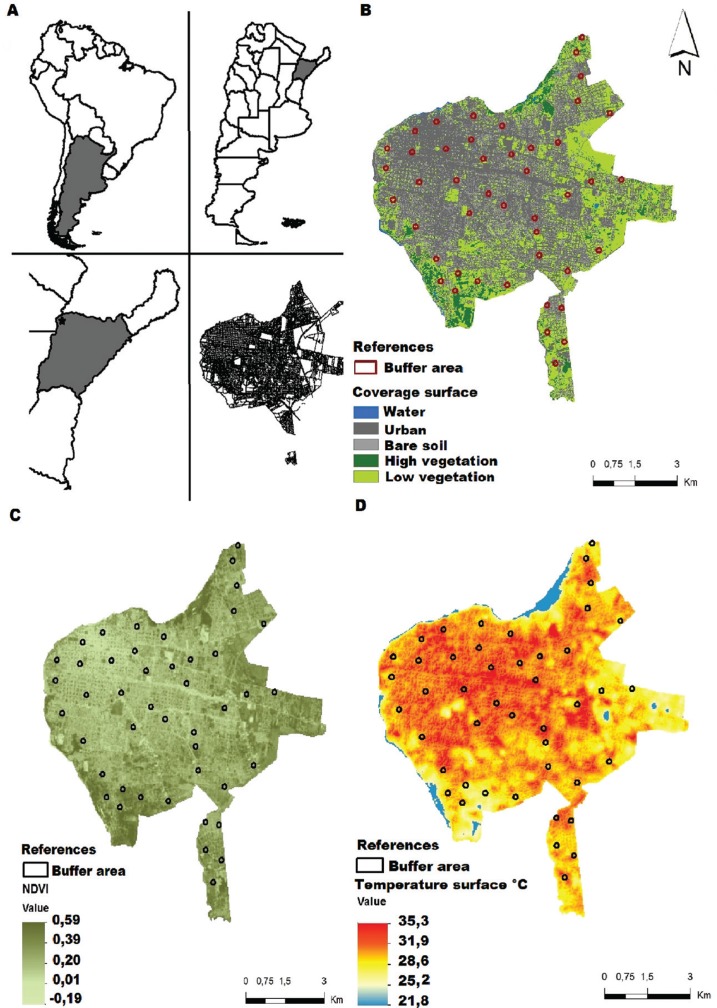
: (A) study area; (B) land coverage map of Corrientes derived from a supervised classification of SPOT 5 images; (C) normalised difference vegetation index map of Corrientes derived from band algebra of LANDSAT 8 images; (D) land surface temperature map of Corrientes derived from band algebra of LANDSAT 8 images.

*Study area* - The study was conducted in the city of Corrientes, northeastern Argentina (27º28’08”S, 58º49’50”W). It is located on the west shore of the Paraná River, in the humid Chaco eco-region. It has a population of 328,868 inhabitants who are distributed over an area of approximately 60 km^2^ (Fig. 1A). As with most of the cities in the region, Corrientes has a complex and heterogeneous urban landscape with a downtown of high buildings and human densities and peri-urban neighbourhoods with mixed patches of houses and green areas.

*Entomological sampling* - In order to obtain a representation of phlebotomine abundance at its most favourable time and to minimise the impact on abundance variability due to daily climatic changes, entomological sampling was carried out between December 20th and 23rd, 2013. The city was stratified according to infrastructure and household density into urban (continuous building and access to all basic services with more than 30 households per hectare), peri-urban (discontinuous building and access to some basic services with 10 to 30 households per hectare) or “rural islands” (isolated buildings and access to some basic services with less than 10 households per hectare) embedded in a peri-urban area. In each stratum, we selected 15 domestic units according to the “worst scenario” criterion (Feliciangeli et al. 2006) for a total of 45 sampled sites. Phlebotominae were captured in peri-domestic environments with REDILA-BL minilight traps (Fernández et al. 2015) placed 1.5 metres above the ground running from approximately 5:30 pm to 7:30 am the following day for three consecutive rainless nights. The captured phlebotominae were diaphonised in lactophenol and then identified in MO 40X using keys according to the methods described by Galati (2003). *Evandromyia cortelezzii* and *Evandromyia sallesi* females were named as *Evandromyia cortelezzii-sallesi* complex, since females are indistinguishable by morphology.

*Micro-habitat variables* - Variables such as drinking water supply, presence of farmyard animals such as hens and/or pigs, asphalt streets (as binary variables) and the number of dogs in the house were obtained by a household survey and direct observation. In addition, the sampling sites were classified according to the area of the plot where the domestic unit is located and included category 1 (greater than 400 m^2^), 2 (between 200 and 400 m^2^) and 3 (smaller than 200 m^2^).

*Macro-habitat variables* - Environmental variables were obtained from multispectral satellite images. A 10-m resolution SPOT 5 J image (November 23, 2013) composed of four spectral bands, namely green (0.50-0.59 nm), red (0.61-0.68 nm), near infrared (0.78-0.89 nm) and infrared (1.58-1.75 nm), was used to generate the supervised classification of the terrestrial coverage. The following five classes of coverage were identified as possible explanatory variables: urban, bare soil, water, high vegetation and low vegetation. The control points of each class were validated by Google Earth^©^ and field confirmation; the constructed confusion matrix showed an overall accuracy of 78.95% and a Kappa coefficient of 0.74. Then, the macro-habitat landscape variables at the urban focus scale were defined as the percentage of each cover class in a buffer area of 80 m around the insect collection site (Fig. 1B). The NDVI and land surface temperature (LST*)* were computed using LANDSAT 8 multispectral image band algebra (December 23, 2013) using the red and near infrared band (NDVI) and the thermal infrared band (LST) (Fig. 1C-D). The average values of the variables were estimated in the 80-m buffer around the entomological sampling sites.

The images were provided by the National Commission on Space Affairs (CONAE). Vector layers and cartographic outputs were generated using Quantum GIS desktop software v.2.6.1 Brighton, and the image processing was carried out with the software Envi 5.1.

*Statistical analysis* - To perform the statistical analysis, we employed generalised linear models using the *MASS* package of the statistical software *R* v.3.3.1 (R Core Team 2016). We constructed a model with a binomial response containing all recorded variables to evaluate the association of the variables with the presence/pseudoabsence of *Lu. longipalpis*. The ‘abundance’ of *Lu. longipalpis* was defined as the cumulative capture during the three consecutive nights of sampling. The following three models with negative binomial response and logarithm link function (to control overdispersion of data) were constructed to evaluate the degree of association of the independent variables with *Lu. longipalpis* abundance (Zuur et al. 2009): a model with micro- and macro-habitat variables, a model with macro-habitat variables and a model with microhabitat variables. The urban coverage and low vegetation variables were eliminated from the macro-habitat variables due to high values of the variance inflation factor (VIF of 233.95 and 7.31 respectively), which were computed using the *car* package for *R* (Fox & Weisberg 2011).

All models were simplified by stepwise elimination of nonsignificant terms using the akaike information criterion adjusted for low sample size (AICc) (Johnson & Omland 2004) using the *MuMIn* package in *R* (Barton 2016). The terms that did not decrease the AICc by at least two units were not retained in the model. The selection of the resulting models was made taking into account the AICc, the ΔAICc and the weights of the tested models. Those with lower AICc values and greater weights were considered as the best models of the set. The model.sel command of *MuMIn* was used to compare the models with each other and to determine which had the highest relative weight (Burnham & Anderson 2002, Johnson & Omland 2004). Spatial autocorrelation was verified using Moran’s I autocorrelation coefficient. Estimates, standard errors (SE) and confidence intervals (CI) (with 95% CI) were calculated by bootstrap of 1000 replications using the R *boot* package (Canty & Ripley 2014).

## RESULTS

**Fig. 2:**
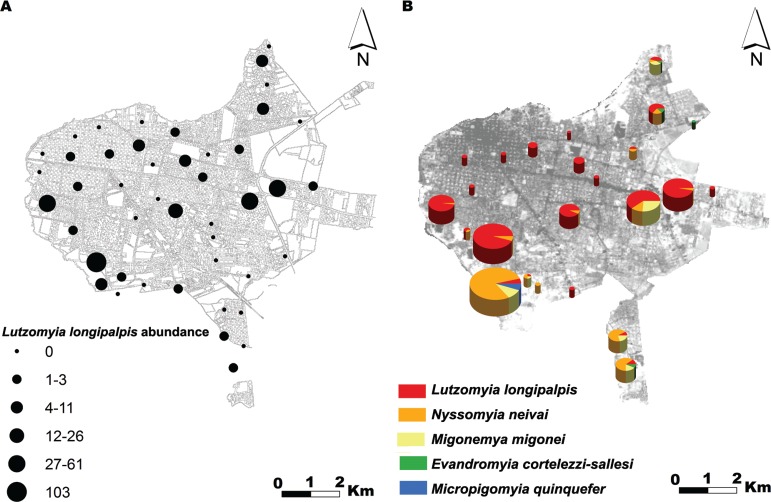
: (A) distribution and abundance of *Lutzomyia longipalpis*; (B) distribution and abundance of phlebotominae, Corrientes, December 2013.

*Entomological results* - We captured 609 phlebotominae representative of the following five species: *Lu. longipalpis*, *Nyssomyia neivai*, *Migonemyia migonei*, *Micropigomyia quinquefer* and *Ev. cortelezzi*-*sallesi* complex (Table I). The most prevalent species, *Lu. longipalpis,* showed a heterogeneous distribution in space (Fig. 2A), with abundances that ranged between 1-7 individuals (15 sites), 8-26 individuals (five sites), and 46-103 individuals (two sites). The spatial distributions of *Ny. neivai*, *Mg. migonei*, *Mi. quinquefer* and *Ev. cortelezzi-Ev. sallesi* were bound to the sites located in the margins of the city connected with vegetation patches (Fig. 2B).

**TABLE I:** Phlebotominae fauna by species captured in Corrientes city, December 2013

Species	Male	Female	Male/female ratio	Total	Relative abundance	Positive sites (%)
*Lutzomyia longipalpis*	266	75	3.55	341	55.99	22 (48.89)
*Nyssomyia neivai*	181	31	5.83	212	34.81	14 (31.11)
*Migonemyia migonei*	26	15	1.73	41	6.74	6 (13.33)
*Micropigomyia quinquefer*	2	7	0.28	9	1.48	1 (2.22)
*Evandromyia cortelezzii-sallesi*	0	6	-	6	0.98	5 (11.11)

Total	475	134	3.54	609	100	-

*Macro-habitat variable modelling* - Urban coverage accounted for 47.4%, bare soil 26.4%, low-density vegetation 22.5% and high-density vegetation 3.7% of the total habitat coverage. The NDVI values of the buffer areas ranged from 0.048 to 0.425 with an average of 0.260 [standard deviation (SD) = 0.082]. The LST values of the buffer areas were on the order of 28.84ºC and 32.57ºC with an average of 31.02ºC (SD = 0.88).

As a result of the stepwise simplification of the models, a set of three models was obtained to predict *Lu. longipalpis* accumulated abundance (negative binomial response models) and one to assess its presence (binomial response model) (Table II).

**TABLE II:** Simplified generalised linear models (GLM) to describe *Lutzomyia longipalpis* abundance and presence in Corrientes city, sorted by weight and Δakaike information criterion (ΔAICc)

Model type	Model	Explanatory variables	AICc	ΔAICc	Weight
Negative binomial for abundance	Micro and macrohabitat	Bare soil + NDVI + farmyard animals	203.82	0	0.854
	Microhabitat	Farmyard animals	207.49	3.66	0.137
	Macrohabitat	NDVI	213.82	10	0.006
	Null negative binomial	1	214.69	10.86	0.004
Binomial for presence	Micro and macrohabitat	Farmyard animals	61.38	0	0.823
	Null binomial	1	64.45	3.07	0.177

NDVI: normalised difference vegetation index.

The simplified model that contained micro- and macro-habitat variables to explain the abundance of *Lu. longipalpis* received the highest score with a weight of 0.854. Together with the simplified model of micro-habitat variables, which contained farmyard animals as the only explanatory variable, the variables were responsible for 99.1% of the weight. The importance of the explanatory variables that composed the models was evaluated and reported in decreasing order of importance: farmyard animals, NDVI and bare soil coverage.

Bootstrap estimates of the coefficients, SD and CI (95% CI) are presented in Table III. Only the variables NDVI and farmyard animals were significant in explaining the accumulated abundance of *Lu. longipalpis* in Corrientes city. The Moran’s I index of spatial autocorrelation was 0.039 (p = 0.59), which described a pattern of complete spatial randomness.

**TABLE III:** Micro and macro habitat variables negative binomial generalised linear models (GLM) of *Lutzomyia longipalpis* abundance: estimates, standard errors (SE) and confidence intervals (CI) by bootstrap of 1000 replications

	Estimate	SE	CI lower limit	CI upper limit
Intercept	-0.93802	0.63066	-1.9311	1.1087
Bare soil	0.73648	0.43975	-0.1279	1.5878
NDVI	-1.67021	0.67154	-3.168	-0.362
Farmyard animals	4.97687	1.27531	2.107	7.563

NDVI: normalised difference vegetation index.

## DISCUSSION

In this study, the implications of environmental factors in the spatial distribution of *Lu. longipalpis* in a subtropical city where an established VL transmission cycle was recently confirmed were analysed. *Lu. longipalpis* was present in 48.9% of the sampled sites, which was a much higher percentage than that recorded by Salomón et al. (2008) for the same study area (24.2%) and similar to that described in other cities of the region, which confirmed species adaptation to the anthropised context (Costa et al. 2005, Santini et al. 2015). The relative abundance (56%) with respect to other phlebotominae species was lower than that found by de Oliveira et al. (2006) in 2005 (92.22%) in Campo Grande, MS, Brazil. However, these authors reported a relative abundance of *Lu. longipalpis* of 8.97% only five years earlier when it was first recorded by de Oliveira et al. (2000). *Lu. longipalpis* was found in Corrientes in 2008 for the first time, so the relative abundance of this species in this newly colonised city is likely to be increasing. The urban spatial distribution of the *L. infantum* vector showed a heterogeneous pattern similar to that described by Fernández et al. (2010) in the city of Posadas with sites of high abundance within a matrix of low abundance. This pattern may be revealing a meta-population behaviour for *Lu. longipalpi*s with source populations capable of colonising other areas of the city as described by Fernández et al. (2013) and de Andrade et al. (2014). The modelling revealed an inverse association between NDVI and *Lu. longipalpis* abundance. Areas with low NDVI may provide few shelter sites for the vector; nevertheless, these areas have clustered domestic animals, meaning abundant organic matter on the soil, concentrated blood-meal sources, and dwellings or a small number of trees as alternative refuges for phlebotominae. These determinations are similar to those observed by Bavia et al. (2005), who suggested that most vectors are found in areas of low NDVI in the state of Bahia, Brazil, but contradict the work of de Oliveira et al. (2012), who observed a positive association between NDVI and the number of collected specimens in a study conducted in Campo Grande, MS, Brazil. However, the latter paper reported a correlation coefficient for both variables that was too low to be considered a true association. In the case of de Andrade et al. (2014), no correlation was found between NDVI and the number of collected sand flies. However, it must be taken into account that our best model contains mixed variables (NDVI and farmyard animals) that act together to explain the distribution of *Lu. longipalpis* abundance and that neither present a significant correlation with the abundance of sand flies when they are considered individually.

The sites with the greatest biological diversity (3-5 species) showed NDVI values that exceeded 0.324. The Spearman correlation test showed a weak association between the NDVI and the Shannon-Wiener biodiversity index for each site (r = 0.47, p = 0.001). This means that at low NDVI values, phlebotomine species richness decreases sharply, which could imply lower interspecific competition between *Lu. longipalpis* and other phlebotominae in low NDVI areas. The variable that showed the greatest weight in explaining both the abundance and the presence of *Lu. longipalpis* was farmyard animals. The association between the presence and abundance of vectors with the presence of farm animals in the peri-domestic environment is in agreement with observations from most other authors (Lainson & Rangel 2005, Fernández et al. 2010, Costa et al. 2013).

The lack of association between *Lu. longipalpis* and LST could be due to the small size of the covered area (60 km^2^) in the present work, which has a low thermal amplitude among sites, and to the fact that no entomological data have been analysed over time taking into account the temperature variations in each site. In a longitudinal study in India, Palaniyandi et al. (2014) emphasised the predictive importance of the LST associated with NDVI in sand fly population dynamics.

The bias due to trap attractiveness or the representativeness of a three-night capture protocol could not be discarded. Therefore, we prefer to state the ‘zero’ result as pseudo-absence rather than as absence of phlebotominae at these sites. On the other hand, to generate strategies that could be used by control program agents, these approaches should have manageable logistics, and a three-night light-trap sampling is a standard protocol used both by programs and researchers. Hence, the results presented here should at least be interpreted as the identification of the ‘main sites and hot spots’.

**Fig. 3:**
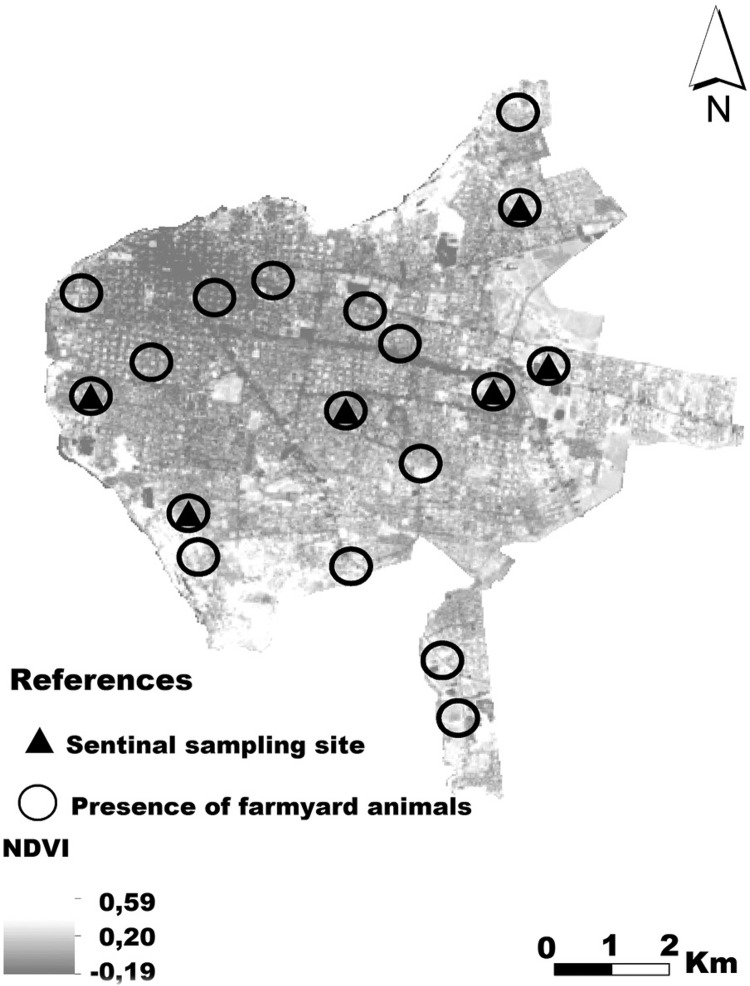
: selection of sentinel sites for time monitoring of the population dynamics of *Lutzomyia longipalpi*s as a risk indicator. Based on the presence of farmyard animals, normalised difference vegetation indices between 0.200 and 0.334 and sites with abundance greater than 10 individuals.

In conclusion, the macro-habitat variables measured through the processing of Remote Sensing products, in association with land-surveyed micro-habitat variables, were significant in explaining the spatial distribution of *Lu. longipalpis* abundance in an urban scenario and could be used to identify priority areas for vector surveillance as shown in an example for Corrientes in Fig. 3. Considering that eight years ago the presence of the vector was not known in Corrientes, and five years later it became the dominant species and many times the only one in anthropised areas, these factors become significant in the short term to establish sentinel sites for entomological surveillance.

The selection of sentinel sites for *Lu. longipalpis* based on RS data instead of a wide city sampling coverage would increase surveillance sensitivity, effectiveness of focused interventions on vector source populations (where and when), and efficiency of both the surveillance and control activities.

The cities in the region are characterised by a complex urban background where a demographic centre generally has infrastructure with access to basic services as opposed to the peri-urban sectors where not all households can access public services. The results presented here also support the hypothesis that this urban complex, which is associated with unplanned urbanisation, is a factor that increases the risk of VL transmission since this favours the abundance of *Lu. longipalpis* (Rangel & Vilela 2008). The particular characteristics of these unprotected sectors could lead to even more detrimental results for the health of the inhabitants in case of a leishmaniasis outbreak.

An ongoing two-year monthly trapping study is being carried out to validate these models over time using additional approaches that better represent the degrees of ‘environmental fitness’ for the vector and in order to analyse the association between variables such as LST temporal evolution with *Lu. longipalpis* abundance.
